# Abyssal deposit‐feeding rates consistent with the metabolic theory of ecology

**DOI:** 10.1002/ecy.2564

**Published:** 2019-01-02

**Authors:** Jennifer M. Durden, Brian J. Bett, Christine L. Huffard, Henry A. Ruhl, Kenneth L. Smith

**Affiliations:** ^1^ Ocean and Earth Science National Oceanography Centre University of Southampton Waterfront Campus, European Way Southampton SO14 3ZH United Kingdom; ^2^ National Oceanography Centre European Way Southampton SO14 3ZH United Kingdom; ^3^ Monterey Bay Aquarium Research Institute 7700 Sandholdt Road Moss Landing California 95039 USA

**Keywords:** deep sea, echinoderm, grazing, ingestion, invertebrate, megafauna

## Abstract

The Metabolic Theory of Ecology (MTE) posits that metabolic rate controls ecological processes, such as the rate of resource uptake, from the individual‐ to the ecosystem‐scale. Metabolic rate has been found empirically to be an exponential function of whole organism body mass. We test a fundamental assumption of MTE, whether resource uptake scales to metabolism, by examining detritivores accessing a single common resource pool, an ideal study case. We used an existing empirical model of ingestion for aquatic deposit feeders adjusted for temperature to test whether ingestion by abyssal deposit feeders conforms to MTE‐predicted feeding rates. We estimated the sediment deposit‐feeding rates of large invertebrates from two abyssal study sites using time‐lapse photography, and related those rates to body mass, environmental temperature, and sediment organic matter content using this framework. Ingestion was significantly related to individual wet mass, with a mass‐scaling coefficient of 0.81, with 95% confidence intervals that encompass the MTE‐predicted value of 0.75, and the same pattern determined in other aquatic systems. Our results also provide insight into the potential mechanism through which this fundamental assumption operates. After temperature correction, both deep‐ and shallow‐water taxa might be summarized into a single mass‐scaled ingestion rate.

## Introduction

The Metabolic Theory of Ecology (MTE; Brown et al. 2004) posits that metabolic rate controls ecological processes, from the individual‐ to the ecosystem‐scale (Schramski et al. 2015), and provides a potentially valuable numerical framework for the interpretation and modeling of ecological processes. A key element in the link between individuals and the ecosystem is the relative distribution of resource use across body mass classes (White et al. 2007). These concepts, and particularly their underlying causes, remain contentious (Isaac et al. 2013). In the simple, symmetric case of this “energetic equivalence,” resource acquisition and use are approximately equivalent in each size class; metabolism is assumed to scale with individual body mass, and numerical abundance in geometric size classes is approximated by a power function. This simple case may only hold within a single trophic level (Brown et al. 2004), with herbivores, or detritivores, accessing a single common resource pool as good candidates for study. Despite being a fundamental assumption of MTE, few studies test whether such resource uptake scales with metabolism.

Prior to formulation of the MTE, Cammen (1980) produced an empirical model of organic matter ingestion rate (*I*_OM_) for aquatic deposit feeders and detritivores, *I*_OM_ = *kM* ^*b*^, where *k* is a normalization constant, *M* is body mass (g fresh wet mass), and *b* is a mass‐scaling coefficient. Cammen (1980) noted that the mass scaling of ingestion rate was comparable to that of metabolism, suggesting a link to, if not control by metabolic demand, and consequently, a high likelihood of strong temperature effects on ingestion rate. Cammen's equation for *I*_OM_ provides a convenient framework with which to assess ingestion across deposit feeders as it relates to body size and suggests the metabolic implications of this relationship. To incorporate the influence of temperature (Gillooly et al. 2001), we add the Van't Hoff‐Arrhenius relation (e^−*E*/*kT*^). Thus, ingestion rate (*I*, mg C/h) can be expressed as *I* = *i*_o_ *M* ^*b*^ *e* ^−*E*/(*kT*)^, where *i*_o_ is a normalization constant independent of body mass and temperature, *E* is the activation energy of metabolism (0.63 eV), *k* is the Boltzmann constant (8.617 × 10^−5^ eV/K), and *T* is the temperature (K). The most appropriate values for the mass‐scaling coefficient and the activation energy of metabolism are a matter of considerable debate (Isaac and Carbone 2010) and the likelihood of any appropriate single values is in question (Brey 2010). Nevertheless, in a major review of aquatic invertebrates (Brey 2010), the majority have a mass‐scaling coefficient of 0.7–0.8, and an activation energy in the range 0.4–0.8 eV.

The marine invertebrate benthos, particularly those of the deep‐sea floor, are very largely dependent on detrital inputs from terrestrial and pelagic ecosystems (Iken et al. 2001, Durden et al. 2015a) and encompass a very large range of detritivore body masses (0.1 μg to >1 kg fresh wet mass), making them ideal for the study of the scaling of resource uptake with body size. The particularly low supply rate of this sole resource to deep‐sea sediments often necessitates mobile feeding strategies to encounter sufficient resources (Jumars and Wheatcroft 1989); as such, it is critical to carbon cycling in those benthic communities (Durden et al. 2017). Studies of metabolism of invertebrates inhabiting the cold deep‐sea environment have largely centered around respiration (Mahaut et al. 1995, Seibel and Drazen 2007, Hughes et al. 2011) and growth and turnover (McClain et al. 2012), rather than testing behavioral processes in the context of MTE. Deposit‐feeding activity has been estimated for a few deep‐sea taxa using time‐lapse photography, often employing lebensspuren (“life traces”) visible on the soft sediment as an indicator of feeding (e.g., echiurans [Bett and Rice 1993], holothurians [Kaufmann and Smith 1997, Smith et al. 1997], and enteropneusts [Smith et al. 2005]). For a deep‐sea echinoid, this feeding activity appeared to be related to body size and variation in organic carbon flux to the seafloor (Vardaro et al. 2009). However, Jumars and Wheatcroft (1989) noted the lack of predictive models connecting deposit feeding, body size, and food quality for deep‐sea invertebrates, along with in situ measurements to support them, and noted that “there is no in situ measurement of feeding rate in any deep‐sea deposit feeder for comparison with Cammen's empirical findings for shallow water.”

We test whether resource uptake scales to metabolism by examining whether ingestion by abyssal deposit feeders conforms to MTE‐predicted feeding rates. To do so, we estimate the sediment deposit‐feeding rates of large abyssal invertebrates at two sites using time‐lapse photography and relate those rates to body mass, environmental temperature, and sediment organic matter content using the mathematical framework outlined above. Finally, we assess whether the temperature‐corrected deposit feeder ingestion rate can be summarized by a simple allometric relationship consistent with that of Cammen (1980) and MTE generally.

## Method

Our data were drawn from two abyssal sites: (1) Porcupine Abyssal Plain in the northeast Atlantic (PAP; 48°50′ N, 16°30′ W, 4850 m water depth; Hartman et al. 2012) and (2) Station M in the northeast Pacific (34°50′ N, 123°06′ W, 4100 m water depth; Smith et al. 2013). At both sites, we deployed time‐lapse cameras to record the presence and activity of large invertebrates (megabenthos). At PAP, a seafloor area of 0.7 m^2^ was monitored at 8‐h intervals for a total of 604 d (1,812 images); at Sta. M, 9.5 m^2^ was assessed at 1‐h intervals for 536 d (12,866 images). Details of the camera deployments and their technical specifications are provided in Appendix S1: Table S1. Image measurements (pixels) were processed to produce corresponding physical dimensions following trigonometric perspective correction (Wakefield and Genin 1987), using the Video Annotation and Reference System (Schlining and Stout 2006), calibrated to the optical geometry of the two camera systems.

We estimated deposit feeding for 15 different taxa, by measuring the creation rates of their associated traces (lebensspuren; see, e.g., Przeslawski et al. 2012). Creation rates were calculated from estimated area and elapsed time; where traces were begun and completed between successive images, creation time was set to half the image interval. Total tracked area was calculated in the case of holothurians that produced continuous lebensspuren. Asteroids typically transit the seabed rapidly between feeding locations, for these taxa only the feeding trace area was calculated. Similarly, echinoids and enteropneusts produce visible tracks while transiting the seabed slowly (and feeding), then relocate quickly to another feeding site. In these cases, only the presumed feeding traces were estimated. For surficial deposit feeders that do not leave an obvious trace (e.g., Elpidiidae spp. at the PAP and *Abyssocucumis abyssorum* and *Oneirophanta mutabilis* at Sta. M), a linear tracking rate was calculated and the areal trace creation rate estimated using the anterior body width as an assumed track width (see, e.g., Bett et al. 2001). Only those specimens in view for at least three successive images were used for tracking rate estimation.

Specimen fresh wet masses were primarily estimated using the length–mass relationships detailed by Durden et al. (2016); where species‐specific conversions were not available, the nearest morphological equivalent was used. An additional species‐specific relationship was developed for *A. abyssorum* (preserved wet mass [g] = 0.0002 × length [mm]^2.57^, *R* ^2^ = 0.94; converted to fresh wet mass using Durden et al. 2016). Where such conversions were not available, individual fresh wet masses were estimated as follows: (1) echinoid test volume was calculated as a regular tetrahedron, tissue volume estimated as 25% of test volume (Ebert 2013), and converted to fresh mass assuming a density of 1 g/cm^3^; (2) enteropneust fresh wet mass was estimated from body width and mean body length (from Smith et al. 2005), and computed using the body geometry method of Jones et al. (2013); and (3) echiuran fresh wet mass was estimated as the geometric mean of trawl‐caught specimen preserved wet mass, converted to fresh wet mass (per Durden et al. 2016).

Ingestion was estimated by taxon (*t*) and site (*x*) as *I*_*tx*_ = *R*_*tx*_ *S*_*t*_DC_*x*_, where *I* is ingestion rate (mg C/h), *R* is the geometric mean tracking rate (cm^2^/h), *S* is the sediment thickness ingested (cm), *D* is the sediment bulk density (g dry mass/cm^3^), and *C* is site‐specific sediment organic carbon content (mg C/g). *S*_*t*_ was based on field and laboratory observations and data (Table 1; Billett 1991, Roberts et al. 2000, Vardaro et al. 2009). *D* for both sites was set at 0.55 g/cm^3^, a typical value for the PAP (Rabouille et al. 2001). *C* for the PAP site (3.75 mg organic C/g dry mass) was based on recent samples (Durden et al. 2017), while that for Sta. M (17.5 mg organic C/g dry mass) was derived from the data of Smith et al. (2001). For temperature correction, the environmental temperatures employed were 2.6°C for PAP (Hall et al. 2007) and 1.5°C for Sta. M (Bauer et al. 1998), consistent with annual climatological means in the World Ocean Atlas 2013 (Locarnini et al. 2013). The Cammen (1980) data set was also assessed, with organic matter content converted to total organic carbon using a factor of 2 (Bader 1954), and a uniform temperature of 15°C, based on Cammen's criteria.

**Table 1:** Parameters for estimation of ingestion at the Porcupine Abyssal Plain (PAP) and Station M (Sta. M) study sites

Site	Group	Taxon	*S*_*t*_ (cm)	*n*	Tracking rate (cm^2^/h)			Individual fresh wet biomass (g)		
					Mean ± SD	Range	Geometric mean (*R*_*t*_)	Mean ± SD	Range	Geometric mean (*R*_*t*_)
PAP	H	Elpidiidae sp.	0.1	9	5.4 ± 6.4	1.5–21.4	3.5	12 ± 17	1–54	6
PAP	H	*Oneirophanta mutabilis*	0.1	13	27.6 ± 15.5	14.3–73.8	24.8	80 ± 48	28–190	69
PAP	H	*Psychropotes longicauda*	0.5	5	34.0 ± 10.4	21.5–44.9	32.7	733 ± 592	33–1360	418
PAP	H	*Molpadiodemas villosus*	1	4	17.5 ± 12.6	6.7–34.1	14.3	898 ± 662	223–1783	700
PAP	A	*Dytaster grandis grandis*	1	1	1.6	–	1.6	45	–	45
PAP		Echiura	0.1	7	2.0 ± 1.3	0.1–4.4	1.5	3	–	3
PAP		All observed tracking		39	17.0 ± 16.2	0.1–73.8	8.7			
Sta. M	H	*Abyssocucumis abyssorum*	0.1	21	38.7 ± 40.5	4.9–160.8	26.3	154.3 ± 106.4	27.8–427.8	125.0
Sta. M	H	*Benthothuria* sp.	0.1	1	12.6	–	12.6	61.3	–	61.3
Sta. M	End	*Cystechinus* sp.	0.01	5	21.5 ± 9.8	9.5–33.0	19.5	2.7 ± 1.1	1.8–4.4	2.5
Sta. M	End	*Echinocrepis* sp.	0.01	13	21.5 ± 7.8	1.9–27.4	6.8	0.9 ± 0.7	0.2–2.8	0.7
Sta. M	H	*Oneirophanta mutabilis*	0.1	9	80.8 ± 51.2	23.7–162.7	66.6	557.7 ± 503.3	215.5–1872.3	452.7
Sta. M	H	*Paelopatides* sp.	0.5	3	26.6 ± 14.0	13.4–41.3	24.1	472.4 ± 270.3	283.8–782.0	427.2
Sta. M	A	*Pseudarchaster* sp.	1	10	78.7 ± 88.0	0.46–211.5	21.9	38.4 ± 15.9	20.7–58.1	35.6
Sta. M	H	*Pseudostichopus mollis*	1	1	7.4	–	7.4	2141.9		2141.9
Sta. M	H	*Psychropotes longicauda*	0.5	2	110.2 ± 89.1	47.3–173.2	90.5	3745.7 ± 647	3288.0–4203.4	3717.7
Sta. M	H	*Stichopus* sp.	1	1	86.8		86.8			1362.9
Sta. M	Ent	*Tergivelum* sp.	0.1	2	3.1 ± 0.4	2.8–3.4	3.1	25.2 ± 7.5	19.9–30.5	24.7
Sta. M		All observed tracking		69	46.2 ± 57.2	0.5–211.5	21.7			

*Notes:* Parameters are sediment thickness ingested by taxon *t* (*S*_*t*_), seafloor tracking rates (means calculated on an individual specimen basis), and individual fresh wet biomass determined from time‐lapse photographs (see Method for taxon‐specific methodologies). Taxon groups are A, Asteroidea; H, Holothuroidea; End, Echinoidea; Ent, Enteropneusta.

For analysis, rate data were converted to a common temperature of 2°C (see, e.g., Gillooly et al. 2001); linear regressions of temperature‐corrected ingestion as ln(*I × e* ^(*E/kT*)^) vs. wet mass as ln(*M*) were fitted using the base function lm and the relationship tested with ANOVA in R (R Core Team 2015), *F* value results are reported on the basis of Type II sum of squares calculations. A general linear model of data from PAP, Sta. M, and Cammen (1980) and ANCOVA were used to assess the influence of body mass (g fresh wet mass) and site on ingestion rate.

## Results

Deposit‐feeding rates, ingestion, and fresh wet masses were estimated for 39 individuals at PAP and 69 individuals at Sta. M (Table 1), covering 15 morphotypes, two of which were present at both study sites (*Oneirophanta mutabilis*,* Psychropotes longicauda*; Table 1). Tracking varied 0.1–212 cm^2^/h, and body mass 0.2–4203 g fresh wet mass; the lowest tracking rates were recorded for enteropneusts, echiurans, and small holothurians, and highest for large holothurians.

**Figure 1:**
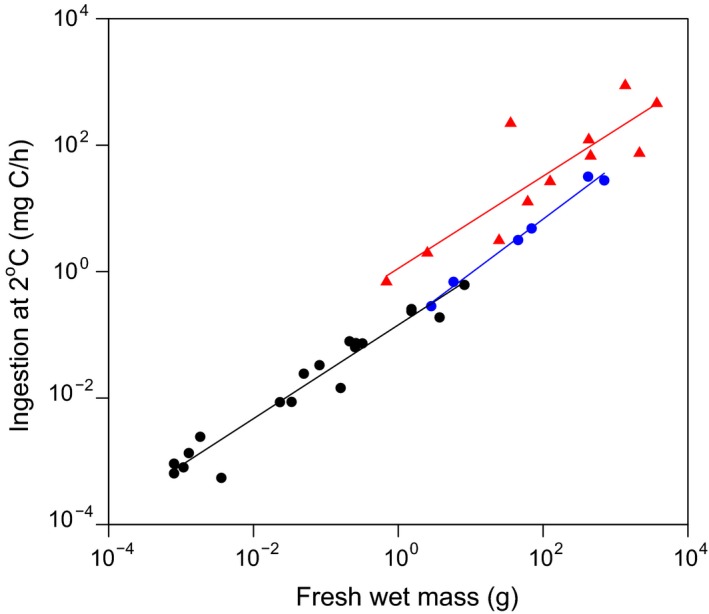
Relationships between individual ingestion rate corrected to 2°C and individual fresh wet biomass computed as geometric means per taxon by site, where ingestion is estimated from areal tracking rates, ingested sediment thickness, sediment bulk density, sediment organic carbon content, and local temperature. Data and linear regressions from Porcupine Abyssal Plain are in blue, from Station M in red, and from Cammen (1980) in black.

The linear relationship between the natural logarithms of ingestion by taxon and fresh wet mass was significant. At the PAP, ingestion scaled with individual wet mass (*F*_1,4_ = 333.1, *P* < 0.0001; *R* ^2^ = 0.99; Fig. 1) with a slope of 0.86 (95% CI 0.73–0.99) and intercept of −2.04 (95% CI −2.61 to −1.46). At Sta. M (*F*_1,9_ = 26.6, *P* < 0.001; *R* ^2^ = 0.73), the slope was 0.73 (95% CI 0.41–1.05) and intercept was 0.12 (95% CI −1.60 to 1.83). When data from both abyssal sites were combined, the regression of ingestion with individual wet mass (*F*_1,15_ = 41.6, *P* < 0.0001; *R* ^2^ = 0.74) resulted in a slope of 0.81 (95% CI 0.54–1.07) and intercept of −0.82 (95% CI −2.17 to 0.53). When Cammen's data were incorporated (*F*_1,34_ = 569.8, *P* < 0.0001, *R* ^2^ = 0.94), the slope was 0.88 (95% CI 0.80–0.95) and intercept was −1.34 (95% CI −1.69, −1.00). Subsequent ANCOVA of body mass and site on ingestion rate, yielded a statistically significant model (*F*_3,32_ = 963.2, *P* < 0.0001), where the influence of site was significant (*F*_2,32_ = 12.74, *R* ^2^ = 0.97, *P* < 0.0001), driven by the Sta. M results (*t* = 4.81, *P* < 0.001). Full outputs from these various statistical models are provided as Appendix S2.

## Discussion

Resource uptake scaled with metabolism in the abyssal deposit feeders we studied; our estimates of organic matter ingestion rate (corrected for environmental temperature) followed the basic predictions of MTE and were consistent with the allometric model proposed by Cammen (1980) for shallow marine deposit feeders and detritivores. The mass‐scaled ingestion rate of the deep‐sea taxa presented here exhibited a mass‐scaling power of 0.81, with 95% confidence intervals that encompass the predicted value of 0.75, and the alternative value of 0.66. Our results also provide some insight into the potential mechanism through which energetic equivalence (White et al. 2007) might operate. Energetic equivalence remains a controversial topic, and energy flux is not typically measured in studies reporting apparent energetic equivalence (Isaac et al. 2013). We have estimated energy acquisition rate, though we are reliant on other studies to estimate corresponding metabolic rates.

Our estimate of the mass‐scaling exponent of individual organic matter ingestion rate, 0.81, is comparable to that of the majority range of aquatic invertebrate metabolism (0.7–0.8; Brey 2010) and studies of deep‐sea rates across a range of taxa (0.6–0.8; Mahaut et al. 1995, Seibel and Drazen 2007, Hughes et al. 2011, McClain et al. 2012). Similarly, the apparent lack of evidence for a differentiation of shallow‐water and deep‐sea rates, when corrected for environmental temperature, is consistent with prior studies (e.g., Childress and Thuesen 1992, Seibel and Drazen 2007, McClain et al. 2012).

In employing geometric mean values of individual body mass and ingestion rate by taxon (and site) in our analyses, we have necessarily disregarded potential systemic variation in these parameters during the period of measurement, and similarly any within‐taxon and within‐environment variation related to behavior and natural history (Jumars and Wheatcroft 1989). We recorded appreciable ranges in mass‐specific bulk sediment ingestion rates for holothurians at PAP (11–34 mg·h^−1^·g fresh wet mass^−1^) and Sta. M (2–35 mg·h^−1^·g fresh wet mass^−1^), which may incorporate temporal variations (e.g., Vardaro et al. 2009). Similar variability has been noted in holothurians (Roberts et al. 2000) and shallow‐water invertebrates (Cammen 1980) and, with values overlapping those of our abyssal sites.

Intra‐annual variations in the quantity of organic matter supplied (order of magnitude), and substantial variations in the composition of that organic matter have been recorded at the PAP (Kiriakoulakis et al. 2001, Lampitt et al. 2010). These variations in organic matter supply may be the source of the effect of site on our linear model, in which the intercept of the linear model of the Sta. M data was higher than those of the models of PAP or Cammen data. The data on organic carbon were collected at a different time to the photographic data at Sta. M; if the organic matter content of the PAP site is used as a common value for both abyssal sites, this “site effect” is removed. If measured concurrently, such variations could impact estimated ingestion rates (Cammen 1989, Vardaro et al. 2009), as ingestion may be varied to accommodate changes to the “quality” of this carbon. Deposit feeder natural history introduces additional systematic variation in apparent resource acquisition rates, for example, caching (e.g., Jumars et al. 1990) where resource is sequestered but not ingested, or dietary switching (e.g., Durden et al. 2015b) where only part of resource ingestion is met from detritus. Temporal variation in megafaunal energy use (metabolism) is also likely, for example, to fuel growth and reproduction, both of which have been linked with seasonal detritus flux in some deep‐sea species (Tyler 1988). Temporal fluctuations in growth imply fluctuations in individual biomass. The energy reserve required for reproduction may be more readily acquired during particular seasons; Tyler (1986) observed that vitellogenesis occurred in many deep‐sea echinoderms following the main seasonal input of organic material.

Spatial variation in ingestion may also occur. Local variation in the deposition of detritus on the deep seafloor occurs at landscape scales as a result of topographic features (Turnewitsch et al. 2015, Morris et al. 2016), and at centimeter to meter scales as aggregations of phytodetritus at the seabed (Lauerman and Kaufmann 1998). Deep‐sea deposit feeders may exploit this patchy resource in a manner similar to optimal foraging theory in terrestrial herbivores (Belovsky 1997), a combination of the spatial pattern of resource use and diet selectivity. Deep‐sea holothurians select food particles based on quality and size (Billett 1991, Ginger et al. 2001). Organic matter may also be vertically stratified in abyssal sediments (Santos et al. 1994), with deposit feeders employing different strategies to access particular sediment horizons (Roberts et al. 2000).

Our work suggests that the joint model derived from Cammen (1980) and temperature‐corrected metabolism (e.g., Gillooly et al. 2001) may have significant ecological value in the formulation of a general model of seafloor ecology and biogeochemistry (e.g., Yool et al. 2017), with applicability to developing modeling tools for understanding human impacts in the ocean, or processes across marine environments. The Cammen (1980) relationship has been used extensively in the modeling of rate processes. As such, the use of modeling approaches based on the MTE will likely have increased applicability to other behavioral/physiological processes, based on testing of its assumptions with empirical data presented here. Deviation from MTE may provide a method for life history and niche concepts to be examined in a framework that accounts for the pervasive influence of MTE concepts.
