# Supplementary material for: Abyssal deposit‐feeding rates consistent with the metabolic theory of ecology
Source: Ecology. 2019 Jan 2;100(1):e02564. doi: 10.1002/ecy.2564 (PMC6850628; doi:10.1002/ecy.2564)
Supplement: Supplementary file 1 [file ECY-100-na-s001.pdf]

Supplementary material for:
Durden, J. M., B. J. Bett, C. L. Huffard, H. A. Ruhl, and K. L. Smith. 2018. Abyssal deposit-feeding rates consistent with the Metabolic Theory of Ecology. *Ecology.*

APPROVED

Appendix S1

Table S1. Camera technical and deployment information. At the Porcupine Abyssal Plain (PAP), 49° 00´ N 016° 27´ W, 4850 m water depth, the Bathysnap system was deployed with an Imenco SDS 1210 stills camera (Bett 2003). At Station M, 34° 50´ N 123° 06´ W, 4000 m water depth, the tripod camera system was deployed with a Canon EOS 5D stills camera (Kaufmann and Smith 1997, Sherman and Smith 2009).

	PAP		Station M		
Reference number	JC062-119	JC071-043	Pulse 58	Pulse 59	Pulse 60
Start (hh:mm dd/mm/yyyy)	12:05 21/08/2011	13:49 06/05/2012	19:29 24/05/2011	20:13 20/11/2011	12:59 13 June 2012
End (hh:mm dd/mm/yyyy)	04:41 03/05/2012	12:30 19/04/2013	04:26 18/11/2011	12:25 11/06/2012	11:00 15/11/2012
Image interval (h)	8	8	1	1	1
Usable images	768	1044	4249	4897	3720
Camera height (m)	0.80	0.80	2.30	2.30	2.30
Vertical field of view (°)	26.6	26.6	35	35	35
Horizontal field of view (°)	35	35	50	50	50
Camera tilt below horizontal (°)	30	30	32	32	32
Field of view assessed (m^2)	0.7262	0.7140	9.53	9.53	9.53

Literature Cited

Bett, B. J. 2003. Time-lapse photography in the deep sea. Underwater Technology 25:121-127.

Sherman, A. D., and K. L. Smith. 2009. Deep-sea benthic boundary layer communities and food supply: A long-term monitoring strategy. Deep Sea Research Part II: Topical Studies in Oceanography 56:1754-1762.
