# Supplementary material for: Abyssal deposit‐feeding rates consistent with the metabolic theory of ecology
Source: Ecology. 2019 Jan 2;100(1):e02564. doi: 10.1002/ecy.2564 (PMC6850628; doi:10.1002/ecy.2564)
Supplement: Supplementary file 2 [file ECY-100-na-s002.pdf]

Supplementary material for:
Durden, J. M., B. J. Bett, C. L. Huffard, H. A. Ruhl, and K. L. Smith. 2018. Abyssal deposit-feeding rates consistent with the Metabolic Theory of Ecology. *Ecology.*

APPROVED

Appendix S2

Full statistical outputs of General Linear Models, ANOVA, ANCOVA and regression.
Significance codes: 0 '***' 0.001 '**' 0.01 '*' 0.05 '.' 0.1 ' ' 1

Linear model of PAP ingestion with wet weight

Call:
lm(formula = log(Ingestion) ~ log(Freshww.g))

Residuals:

Amperima	Dytaster.grandis	Echiura	Oneirophanta
0.15925	-0.07937	-0.11849	-0.02559

Pseudostichopus.villosus	Psychropotes.longicauda
-0.25726	0.32146

Coefficients:

	Estimate	Std. Error	t value	Pr(>|t|)
(Intercept)	-2.03758	0.20665	-9.86	0.000594 ***
log(Freshww.g)	0.85768	0.04699	18.25	5.3e-05 ***

Residual standard error: 0.2323 on 4 degrees of freedom
Multiple R-squared: 0.9881, Adjusted R-squared: 0.9852
F-statistic: 333.1 on 1 and 4 DF, p-value: 5.3e-05

95% confidence intervals:

	2.5 %	97.5 %
(Intercept)	-2.6113429	-1.4638254
log(Freshww.g)	0.7272109	0.9881511

ANOVA of PAP ingestion with wet weight

	Df	Sum Sq	Mean Sq	F value	Pr(>F)
log(Freshww.g)	1	17.978	17.978	333.1	5.3e-05 ***
Residuals	4	0.216	0.054		

Supplementary material for:
Durden, J. M., B. J. Bett, C. L. Huffard, H. A. Ruhl, and K. L. Smith. 2018. Abyssal deposit-feeding rates consistent with the Metabolic Theory of Ecology. *Ecology.*

Linear model of Station M ingestion with wet weight

Call:
lm(formula = log(Ingestion) ~ log(Freshww.g))

Residuals:

Min	1Q	Median	3Q	Max
-1.3995	-0.4731	-0.2148	0.1396	2.6794

Coefficients:

	Estimate	Std. Error	t value	Pr(>|t|)
(Intercept)	0.1154	0.7592	0.152	0.882504
log(Freshww.g)	0.7297	0.1415	5.158	0.000597 ***

Residual standard error: 1.227 on 9 degrees of freedom
Multiple R-squared: 0.7472, Adjusted R-squared: 0.7191
F-statistic: 26.6 on 1 and 9 DF, p-value: 0.0005967

95% confidence intervals:

	2.5 %	97.5 %
(Intercept)	-1.6020661	1.832944
log(Freshww.g)	0.4096486	1.049665

ANOVA of Station M ingestion with wet weight

	Df	Sum Sq	Mean Sq	F value	Pr(>F)
log(Freshww.g)	1	40.07	40.07	26.61	0.000597 ***
Residuals	9	13.56	1.51		

Linear model of PAP and Station M ingestion with wet weight

Call:
lm(formula = log(Ingestion) ~ log(Freshww.g))

Residuals:

Min	1Q	Median	3Q	Max
-1.2861	-1.0388	0.0292	0.7204	3.3296

Coefficients:

	Estimate	Std. Error	t value	Pr(>|t|)
(Intercept)	-0.8193	0.6335	-1.293	0.216
log(Freshww.g)	0.8093	0.1255	6.446	1.1e-05 ***

Residual standard error: 1.268 on 15 degrees of freedom
Multiple R-squared: 0.7348, Adjusted R-squared: 0.7171
F-statistic: 41.56 on 1 and 15 DF, p-value: 1.101e-05

95% confidence intervals:

	2.5 %	97.5 %
(Intercept)	-2.1696101	0.531058
log(Freshww.g)	0.5417139	1.076900

Linear model of all three datasets

Call:
lm(formula = log(Ingestion) ~ log(Freshww.g))

Residuals:

Min	1Q	Median	3Q	Max
-1.4735	-0.6248	-0.0946	0.3071	3.6151

Coefficients:

	Estimate	Std. Error	t value	Pr(>|t|)
(Intercept)	-1.34366	0.16812	-7.992	2.59e-09 ***
log(Freshww.g)	0.87617	0.03671	23.870	< 2e-16 ***

Residual standard error: 1.002 on 34 degrees of freedom
Multiple R-squared: 0.9437, Adjusted R-squared: 0.942
F-statistic: 569.8 on 1 and 34 DF, p-value: < 2.2e-16

ANOVA of all three datasets – ingestion with wet weight

	Df	Sum Sq	Mean Sq	F value	Pr(>F)
log(Freshww.g)	1	571.5	571.5	963.18	< 2e-16 ***
Site	2	15.1	7.6	12.74	8.52e-05 ***
Residuals	32	19.0	0.6		

ANCOVA on General Linear Model of ingestion with fresh wet weight and site

Call:
lm(formula = log(Ingestion) ~ log(Freshww.g) + Site

Residuals:

Min	1Q	Median	3Q	Max
-1.45669	-0.30262	-0.02816	0.27531	2.70074

Coefficients:

	Estimate	Std. Error	t value	Pr(>|t|)
(Intercept)	-1.91980	0.22477	-8.541	9.25e-10 ***
log(Freshww.g)	0.74882	0.04769	15.701	< 2e-16 ***
SitePAP	0.30754	0.48569	0.633	0.53110
SiteStaM	1.94542	0.46530	4.181	0.00021 ***

Residual standard error: 0.7703 on 32 degrees of freedom
Multiple R-squared: 0.9686, Adjusted R-squared: 0.9657
F-statistic: 329.6 on 3 and 32 DF, p-value: < 2.2e-16
